# Time Trends of Period Prevalence Rates of Patients with Inhaled Long-Acting Beta-2-Agonists-Containing Prescriptions: A European Comparative Database Study

**DOI:** 10.1371/journal.pone.0117628

**Published:** 2015-02-23

**Authors:** Marietta Rottenkolber, Eef Voogd, Liset van Dijk, Paola Primatesta, Claudia Becker, Raymond Schlienger, Mark C. H. de Groot, Yolanda Alvarez, Julie Durand, Jim Slattery, Ana Afonso, Gema Requena, Miguel Gil, Arturo Alvarez, Ulrik Hesse, Roman Gerlach, Joerg Hasford, Rainald Fischer, Olaf H. Klungel, Sven Schmiedl

**Affiliations:** 1 Institute for Medical Information Sciences, Biometry, and Epidemiology, Ludwig-Maximilians-Universitaet Muenchen, Munich, Germany; 2 Utrecht Institute for Pharmaceutical Sciences, Division Pharmacoepidemiology and Clinical Pharmacology, Utrecht University, Utrecht, the Netherlands; 3 Netherlands Institute for Health Services Research, Utrecht, the Netherlands; 4 Novartis Pharma AG, Basel, Switzerland; 5 Division Clinical Pharmacy & Epidemiology, University of Basel, Basel, Switzerland; 6 European Medicines Agency, London, United Kingdom; 7 Pharmacology Unit, Department of Biomedical Sciences II, School of Medicine and Health Sciences, University of Alcalá, Madrid, Spain; 8 BIFAP Unit, Division of Pharmacoepidemiology and Pharmacovigilance, Spanish Agency for Medicines and Medical Devices, Madrid, Spain; 9 Danish Medicines Agency (Lægemiddelstyrelsen), Medicines Control Division, Copenhagen, Denmark; 10 National Association of Statutory Health Insurance Physicians of Bavaria, Munich, Germany; 11 Medizinische Klinik und Poliklinik V, University Hospital, Ludwig-Maximilians-Universitaet Muenchen, Munich, Germany; 12 Department of Clinical Pharmacology, School of Medicine, Faculty of Health, Witten/Herdecke University, Witten, Germany; 13 Philipp Klee-Institute for Clinical Pharmacology, HELIOS Clinic Wuppertal, Wuppertal, Germany; Bielefeld Evangelical Hospital, GERMANY

## Abstract

**Background:**

Inhaled, long-acting beta-2-adrenoceptor agonists (LABA) have well-established roles in asthma and/or COPD treatment. Drug utilisation patterns for LABA have been described, but few studies have directly compared LABA use in different countries. We aimed to compare the prevalence of LABA-containing prescriptions in five European countries using a standardised methodology.

**Methods:**

A common study protocol was applied to seven European healthcare record databases (Denmark, Germany, Spain, the Netherlands (2), and the UK (2)) to calculate crude and age- and sex-standardised annual period prevalence rates (PPRs) of LABA-containing prescriptions from 2002–2009. Annual PPRs were stratified by sex, age, and indication (asthma, COPD, asthma and COPD).

**Results:**

From 2002–2009, age- and sex-standardised PPRs of patients with LABA-containing medications increased in all databases (58.2%–185.1%). Highest PPRs were found in men ≥ 80 years old and women 70–79 years old. Regarding the three indications, the highest age- and sex-standardised PPRs in all databases were found in patients with “asthma and COPD” but with large inter-country variation. In those with asthma or COPD, lower PPRs and smaller inter-country variations were found. For all three indications, PPRs for LABA-containing prescriptions increased with age.

**Conclusions:**

Using a standardised protocol that allowed direct inter-country comparisons, we found highest rates of LABA-containing prescriptions in elderly patients and distinct differences in the increased utilisation of LABA-containing prescriptions within the study period throughout the five European countries.

## Background

Asthma and chronic obstructive pulmonary disease (COPD) are two of the most prevalent chronic diseases worldwide [1]. Thus, global, evidence-based initiatives were developed to improve and standardise the treatment of both diseases [2, 3]. Despite the wide acceptance of these guidelines, frequent off-label use of respiratory drugs has been reported, especially in children [4, 5]. Long-acting beta-2-agonists (LABA) are a key drug class for the treatment of both COPD and asthma [2, 3]. After the SMART trial [6], the risks of LABA use in asthma patients not receiving inhaled corticosteroids (ICS) were widely discussed [7]. Most recently, several publications have also reported relevant risks for the combination of LABA and ICS in asthma patients [8, 9]. In addition, some data have called into question the benefit-risk profile of LABA in COPD patients [10].

Despite the broad use of LABA-containing drugs and the unsolved issues regarding risks and benefits, there are still only a limited number of drug-utilisation studies that provide detailed data on LABA-containing medications [11, 12]. In particular, carefully conducted, inter-country comparisons of drug treatment characteristics and of populations at risk of overtreatment are scarce [12]. Unfortunately, most available studies compare different types of databases, time periods, etc. limiting the value of these inter-country comparisons to a significant degree [11].

Our goal was to compare the prevalence of LABA-containing prescriptions in seven databases from five different European countries that are part of the collaborative effort known as “Pharmacoepidemiological Research on Outcomes of Therapeutics by a European Consortium” (PROTECT) [13]. To the best of our knowledge, this analysis represents the first study in which the prevalence of LABA-containing prescriptions is optimally compared across different countries by the application of a standardised protocol. Furthermore, we specifically stratified the prevalence of the LABA-containing prescriptions by age, sex, calendar year and recorded indication (“asthma”, “COPD”, “asthma and COPD”).

## Methods

### Data sources

The following seven European healthcare record databases were analysed: Mondriaan–Netherlands Primary Care Research Database (Mondriaan-NPCRD, The Netherlands) [14], Mondriaan–Almere Health Care Group (Mondriaan-AHC, The Netherlands) [15], The United Kingdom Clinical Practice Research Datalink (CPRD, United Kingdom) [16], The Health Improvement Network (THIN, United Kingdom) [17], Computerized Database for Pharmacoepidemiological Studies in Primary Care (BIFAP, Spain) [18], The Bavarian Association of Statutory Health Insurance Physicians database (Bavarian DB, Germany) [19], and the Danish national registries (DKMA, Denmark) [20]. The different databases cover regionally / nationally representative populations of 170,000 to 10.5 million people. The main characteristics of the databases are presented in Table 1 and have been described in detail elsewhere [21].

**Table 1:** Main characteristics of databases.

Database	Country	Population size of DB	Population coverage	Study period	Data source	Prescriptions of specialist included	Drug coding system	Disease coding system	Recording of drug use	Routine data quality checks	Specifics
**Mondriaan—NPCRD** (Mondriaan–Netherlands Primary Care Research Database)	The Netherlands (National)	340,000	2%	2002–2009	Primary care	No	ATC	ICPC	Prescriptions	Yes	-
**Mondriaan—AHC** (Mondriaan–Almere Health Care Group)	The Netherlands (Regional)	170,000	1% of the population of the Netherlands, 90.3% of Almere citizens in 2008	2002–2008	Primary care and pharmacy	Yes	ATC	ICPC	Prescriptions and dispensings	Yes	-
**CPRD** (The United Kingdom Clinical Practice Research Datalink)	UK (National)	> 5 million	7%	2002–2009	Primary care	No	Multilex	Read Codes	Prescriptions	Yes	-
**THIN** (The Health Improvement Network)	UK (National)	3.8 million	6.2%	2002–2009	Primary care	No	Multilex	Read Codes	Prescriptions	Yes	-
**BIFAP** (Computerized Database for Pharmacoepidemiological Studies in Primary Care)	Spain (National)	3.1 million	6.8%	2002–2009	Primary care	No	ATC	ICPC	Prescriptions	Yes	-
**DKMA** (Danish national registries)	Denmark (National)	5.5 million	100%	2002–2009	Database linkage government and health database	Yes	ATC	ICD-10	Prescriptions and dispensings	Yes	-
**Bavarian DB** (The Bavarian Association of Statutory Health Insurance Physicians database)	Germany (Regional)	10.5 million	13% of the population of Germany, 85% of the population of Bavaria	2004–2008	Insurance claims database	Yes	ATC	ICD-10-GM	Claims based on reimbursed prescriptions	Yes	Prescriptions and diagnoses are documented on a quarterly base (no specific date)

ATC: Anatomical Therapeutic Chemical Classification System.

ICPC: International Classification in Primary Care.

ICD-10: International Statistical Classification of Diseases and Related Health Problems (ICD-10).

ICD-10-GM: International Statistical Classification of Diseases and Related Health Problems German Modification.

There is some overlap of doctors’ offices in the UK databases, CPRD and THIN (n = 327 for CPRD and THIN combined, n = 286 for CPRD alone, and n = 168 for THIN alone) [22]. The documented data from the overlapping practices were included in the analyses of each database, and therefore they are not mutually exclusive. There was no overlap between the two Dutch databases. The International Statistical Classification of Diseases and Related Health Problems (ICD-10), the International Classification in Primary Care (ICPC) or the Read Codes were used for coding diagnoses. The Anatomical Therapeutic Chemical classification system (ATC) or Multilex Codes were used for coding drugs.

### Study population and study period

All patients with available data within the period of valid data collection (January 1, 2002—December 31, 2009) were included in this study. In two databases (Bavarian DB and Mondriaan–AHC) the study period differed slightly (2004–2008 and 2002–2008, respectively). Since 2008 was the year with the most recent information available in all of the databases, it was the year selected for the analyses of indication and number of prescriptions per year.

**Exposure definition**. Exposure was defined as at least one prescription containing an inhaled long-acting beta-2-adrenoceptor agonist (LABA; salmeterol or formoterol) including fixed combination drugs (S1 Table) irrespective of any other concomitant medication.

### Statistical analyses

Crude annual period prevalence rates (PPRs) were calculated for all patients with LABA-containing prescriptions (irrespective of indication) by dividing all patients with at least one recorded prescription for a LABA-containing medicine by the total number of people available in each database at midyear. For all PPRs, direct standardisation by age and sex was performed based upon the European standard population in 2008 [23, 24]. 95% Confidence intervals were calculated for crude PPR based on the modified Wald method and for the age-and sex-standardised PRR according to the Spiegelman method [25, 26].

The PPRs were calculated stratifying by age (ten-year age groups) and sex. For the Bavarian database, the youngest age group available was “0–19 years”. For the analysis of the annual number of inhaled LABA-containing prescriptions per patient the following categories were used: “1 prescription”, “2–11 prescriptions”, “12–23 prescriptions”, “24+ prescriptions”. Stratification by indication was performed for the year 2008 for all patients receiving at least one LABA-containing prescription. The indication was defined retrospectively at the date of the last LABA prescription within the study period (2002–2009). This entailed searching the entire study period backwards for medical codes to classify the patients into the following mutually exclusive categories: “asthma”, “COPD”, “asthma and COPD”, and “other and unknown”. Asthma and COPD were defined by ICD-10, ICPC or Read codes (S2–S5 Tables). The category “other and unknown” includes patients with a diagnosis other than asthma or COPD or not having a documented diagnosis. In the DKMA database, the stratification by indication was not performed. Additional crude and standardised annual PPRs were calculated for the strata “asthma”, “COPD” and “asthma and COPD” for each year in the study period. The denominator for these analyses was the number of patients who were available in the respective stratum of each database at midyear. Comparing groups, the chi-square test was used for categorical variables. P-values <0.05 were considered statistically significant.

## Results

### I. All patients with LABA-containing prescriptions (irrespective of indication)

**Fig 1:**
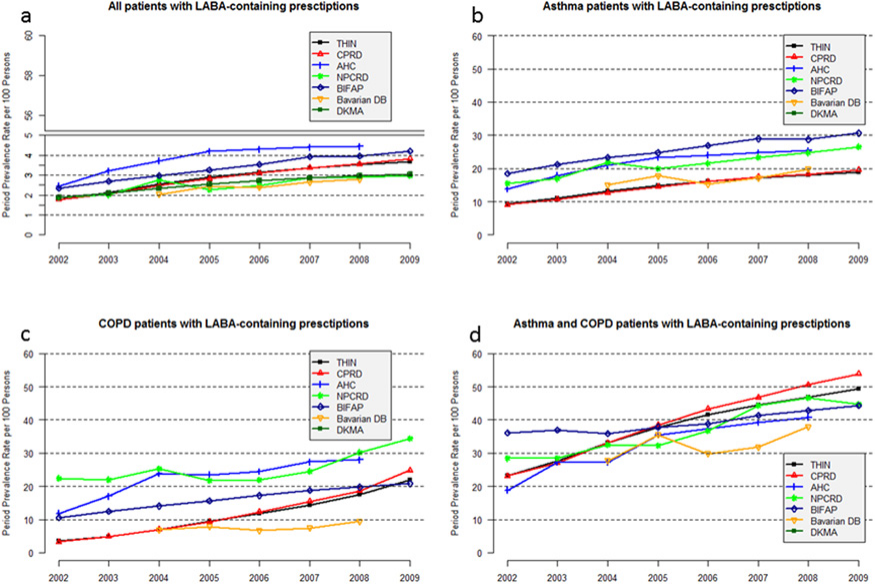
Age- and sex-standardised annual PPR of patients with LABA-containing prescriptions per 100 persons, for all patients with LABA-containing prescriptions (a), asthma patients with LABA-containing prescriptions (b), COPD patients with LABA-containing prescriptions (c), and asthma and COPD patients with LABA-containing prescriptions (d) (AHC: Mondriaan–AHC, NPCRD: Mondriaan–NPCRD).

**Annual period prevalence rates**. The age- and sex-standardised annual PPRs of patients with LABA-containing prescriptions increased for all databases from 2002 onwards (Fig. 1a). The highest percentage increase within the study period was observed in the BIFAP and the CPRD database at 185.1% and 116.3%, respectively. The lowest increase was found in the Mondriaan–NPCRD database at 58.2%.

In 2008, the Mondriaan–AHC database and the BIFAP database had the highest standardised PPR (4.4 per 100 persons and 4.0 per 100 persons, respectively). The lowest standardised PPRs in 2008 were observed in the Bavarian DB and Mondriaan–NPCRD databases (2.8 per 100 persons and 2.9 per 100 persons, respectively) (Table 2).

**Table 2:** Comparison of LABA prescribing in 7 European databases stratified by indication for the year 2008.

		THIN	CPRD	Mondriaan–AHC	Mondriaan–NPCRD	BIFAP	Bavarian DB	DKMA
**Persons with at least one LABA prescription**	Total	128,220	154,505	5,159	8,964	56,144	302,993	153,630
	Asthma	79,540 (62.0%)	96,687 (62.6%)	2,706 (52.5%)	3,529 (39.4%)	25,582 (45.6%)	109,639 (36.2%)	n.a.
	COPD	13,776 (10.7%)	15,703 (10.2%)	962 (18.6%)	2,049 (22.9%)	12,704 (22.6%)	56,489 (18.6%)	n.a.
	Asthma and COPD	30,389 (23.7%)	36,152 (23.4%)	164 (3.2%)	1,447 (16.1%)	2,369 (4.2%)	103,365 (34.1%)	n.a.
	Other or unknown	4,515 (3.5%)	5,963 (3.9%)	1,327 (25.7%)	1,939 (21.6%)	15,489 (27.6%)	33,500 (11.1%)	n.a.
**Total number of persons in the database at mid-year**	Total	3,704,927	4,273,098	141,419	323,175	1,441,009	10,415,393	5,242,117
	Asthma	497,585 (13.4%)	586,707 (13.7%)	12,619 (8.9%)	16,428 (5.1%)	112,562 (7.8%)	569,482 (5.5%)	n.a.
	COPD	40,233 (1.1%)	41,248 (1.0%)	2,075 (1.5%)	4,532 (1.4%)	27,338 (1.9%)	409,825 (3.9%)	n.a.
	Asthma and COPD	45,664 (1.2%)	51,224 (1.2%)	253 (0.2%)	2,496 (0.8%)	3,266 (0.2%)	246,500 (2.4%)	n.a.
**Crude annual PPR per 100 persons (95% CI)**	Total	3.5 (2.9–4.1)	3.6 (3.1–4.2)	3.6 (1.4–8.4)	2.8 (1.4–5.3)	3.9 (3.0–5.0)	2.9 (2.0–4.1)	2.9 (2.5–3.4)
	Asthma	16.0 (13.0–19.5)	16.5 (13.7–19.7)	21.4 (6.4–49.8)	21.5 (7.6–46.2)	22.7 (15.9–31.3)	19.3 (16.2–22.7)	n.a.
	COPD	34.2 (21.5–49.8)	38.1 (24.8–53.3)	46.4 (8.5–89.0)	45.2 (13.6–81.2)	46.5 (29.4–64.4)	13.8 (10.8–17.5)	n.a.
	Asthma and COPD	66.5 (52.0–78.5)	70.6 (56.9–81.3)	64.4 (58.4–70.1)	58.0 (14.3–92.0)	72.5 (24.4–96.3)	41.9 (35.9–48.2)	n.a.
**Standardised annual PPR per 100 persons (95% CI)**	Total	3.5 (3.5–3.5)	3.6 (3.5–3.6)	4.4 (4.3–4.6)	2.9 (2.8–3.0)	4.0 (3.9–4.0)	2.8 (2.8–2.8)	3.0 (2.9–3.0)
	Asthma	18.1 (18.0–18.2)	18.3 (18.2–18.4)	25.5 (24.5–26.4)	24.7 (23.9–25.4)	28.7 (28.4–29.0)	19.8 (19.6–19.9)	n.a.
	COPD	17.5 (16.5–18.5)	18.6 (17.8–19.5)	28.0 (23.1–32.9)	30.3 (25.0–35.5)	19.7 (19.0–20.5)	9.4 (9.3–9.5)	n.a.
	Asthma and COPD	46.8 (45.6–48.0)	50.6 (49.2–52.0)	40.8 (35.9–45.7)	46.7 (42.3–51.0)	42.8 (40.1–45.5)	37.9 (37.7–38.2)	n.a.

LABA: Long-acting beta-2-agonist.

n.a.: not available.

PPR: Period prevalence rate.

CI: Confidence interval.

The PPR of the Mondriaan-AHC database was most affected by the standardisation procedure (3.6 per 100 persons before standardisation, 4.4 per 100 persons after standardisation). Only minor changes were detected in the other databases after standardisation.

**Annual period prevalence rates stratified by age and sex**. Table 3 shows the results for the sex-specific PPRs of patients with LABA-containing prescriptions, stratified by age, for the year 2008. The same patterns were observed in all other years (data not shown). The PPR was higher in men, particularly in males 0–20 years old and males over 70 years old. A continuous increase over the age groups was observed for females until the age of 80 years. For females ≥ 80 years old, a decrease in PPR was noted. The situation was different for males, as their PPR continued to increase and was highest in the age group “≥ 80 years”. In the BIFAP database, the highest PPR was observed among males aged ≥ 80 years, at 16.7 per 100 persons.

**Table 3:** Comparison of annual period prevalence rate (PPR) of LABA-containing prescriptions in 7 European databases stratified by age and sex for the year 2008.

Age group	THIN		CPRD		Mondriaan–AHC		Mondriaan–NPCRD		BIFAP		Bavarian DB		DKMA	
	PPR per 100 persons (95% CI)		PPR per 100 persons (95% CI)		PPR per 100 persons (95% CI)		PPR per 100 persons (95% CI)		PPR per 100 persons (95% CI)		PPR per 100 persons (95% CI)		PPR per 100 persons (95% CI)	
	Male	Female	Male	Female	Male	Female	Male	Female	Male	Female	Male	Female	Male	Female
**0–9 years**	0.9	0.6	0.8	0.6	1.9	1.5	0.6	0.3	0.9	0.6	-	-	0.6	0.4
	(0.5–1.4)	(0.0–3.2)	(0.0–3.2)	(0.0–3.0)	(0.0–34.2)	(0.0–34.8)	(0.0–20.5)	(0.0–21.1)	(0.7–1.1)	(0.2–1.7)			(0.0–2.3)	(0.0–2.1)
**10–19 years**	2.0	1.8	2.2	1.8	2.6	2.2	1.3	1.2	2.3	2.1	1.6*	1.2*	2.0	1.6
	(0.7–4.8)	(0.6–4.7)	(0.9–4.9)	(0.6–4.4)	(0.0–33.4)	(0.0–34.5)	(0.0–20.8)	(0.0–21.6)	(1.4–3.9)	(1.2–3.6)	(1.0–2.6) *	(0.6–2.0) *	(0.9–4.2)	(0.6–3.8)
**20–29 years**	1.6	2.3	1.7	2.1	1.3	2.2	1.1	1.5	2.0	2.4	1.4	1.6	1.2	1.5
	(0.4–4.3)	(0.9–5.2)	(0.5–4.3)	(0.9–4.7)	(0.0–36.0)	(0.0–36.3)	(0.0–20.9)	(0.0–20.1)	(1.3–3.0)	(1.6–3.4)	(0.7–2.7)	(0.9–3.0)	(0.3–3.4)	(0.5–3.9)
**30–39 years**	2.0	2.8	2.2	2.8	1.8	2.7	1.5	1.9	2.1	2.4	1.8	2.1	1.6	1.9
	(0.8–4.7)	(1.3–5.8)	(0.9–4.9)	(1.3–5.4)	(0.0–33.4)	(0.0–32.3)	(0.0–18.7)	(0.0–18.8)	(1.4–3.3)	(1.8–3.4)	(0.9–3.2)	(1.2–3.5)	(0.6–3.6)	(0.8–4.1)
**40–49 years**	2.4	3.6	2.6	3.6	2.6	4.8	2.2	3.2	2.2	3.3	2.1	2.6	1.9	2.7
	(1.1–5.1)	(1.9–6.6)	(1.3–5.2)	(1.6–7.4)	(0.0–30.3)	(0.0–32.4)	(0.0–18.2)	(0.0–19.9)	(1.4–3.3)	(2.4–4.5)	(1.3–3.3)	(1.8–3.9)	(0.9–3.9)	(1.5–5.0)
**50–59 years**	3.4	4.8	3.5	4.7	4.4	6.4	3.3	4.3	3.7	4.3	3.1	3.5	2.8	4.2
	(1.6–6.7)	(2.6–8.4)	(1.8–6.5)	(2.7–8.0)	(0.0–37.4)	(0.0–40.8)	(0.0–22.0)	(0.0–23.6)	(2.6–5.2)	(3.2–5.8)	(2.0–4.8)	(2.4–5.2)	(1.5–5.2)	(2.5–6.9)
**60–69 years**	5.9	6.7	5.8	6.6	6.1	8.6	4.9	5.4	8.0	6.4	5.2	4.8	4.4	5.8
	(3.3–10.3)	(3.9–11.2)	(3.4–9.6)	(4.1–10.5)	(0.0–55.4)	(0.0–57.5)	(0.0–28.6)	(0.0–29.5)	(6.1–10.4)	(4.8–8.5)	(3.6–7.4)	(3.4–6.8)	(2.6–7.3)	(3.7–9.0)
**70–79 years**	8.7	8.3	8.6	8.4	11.9	11.9	8.0	6.9	14.4	8.5	6.9	5.3	8.1	8.6
	(4.7–15.3)	(4.6–14.1)	(5.0–14.4)	(5.0–13.5)	(0.0–74.3)	(0.0–70.3)	(0.0–42.8)	(0.0–38.0)	(11.4–18.0)	(6.5–11.1)	(4.8–9.8)	(3.7–7.5)	(4.7–13.4)	(5.4–13.5)
**≥ 80 years**	9.2	6.4	9.4	6.7	12.1	10.0	8.3	5.4	16.7	8.1	7.4	4.5	8.0	5.6
	(3.7–20.1)	(2.8–13.1)	(4.4–18.2)	(3.5–12.3)	(0.0–85.7)	(0.0–79.3)	(0.0–58.4)	(0.0–42.4)	(12.3–22.2)	(6.0–11.0)	(4.2–12.6)	(2.8–7.1)	(3.5–16.4)	(2.7–10.7)
**Total**	3.1	3.8	3.4	3.8	3.1	4.2	2.6	3.0	4.0	3.8	2.9	2.9	2.6	3.2
	(2.4–4.0)	(3.0–4.8)	(2.7–4.2)	(3.1–4.7)	(0.3–10.8)	(0.9–12.1)	(0.8–6.6)	(1.1–7.0)	(3.6–4.5)	(3.4–4.3)	(2.5–3.4)	(2.5–3.4)	(2.1–3.3)	(2.6–4.0)

LABA: Long-acting beta-2-agonist.

CI: Confidence interval.

*Age group: 0–19 years

**Fig 2:**
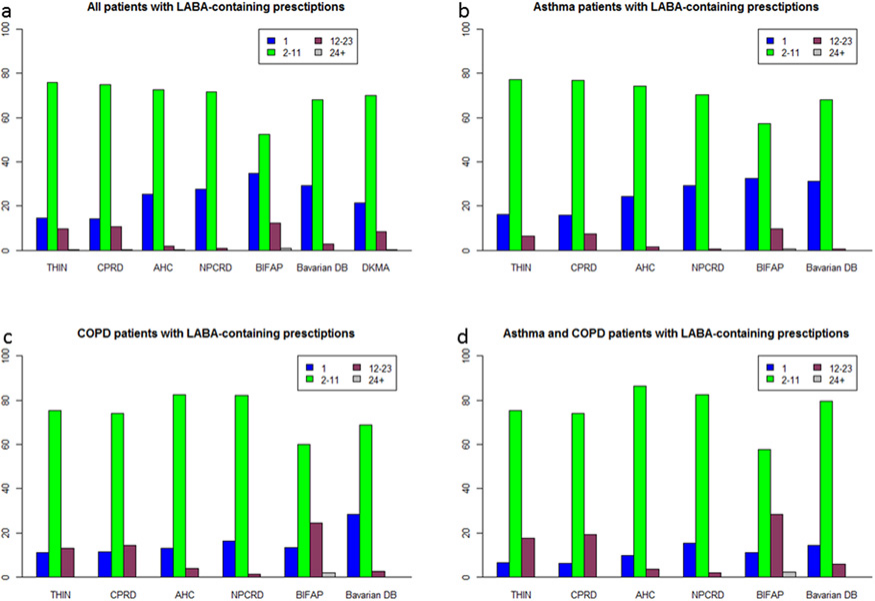
Proportion of patients with “1”, “2–11”, “12–23”, and “24+” LABA containing-prescriptions in 2008, for all patients with LABA-containing prescriptions (a), asthma patients with LABA-containing prescriptions (b), COPD patients with LABA-containing prescriptions (c), and asthma and COPD patients with LABA-containing prescriptions (d) (AHC: Mondriaan–AHC, NPCRD: Mondriaan–NPCRD).

**Number of LABA prescriptions in 2008**. The number of LABA-containing prescriptions per year was significantly different between the database (p<0.0001). In all databases most patients received 2–11 LABA-containing prescriptions per year (between 52.2% and 75.8%). The proportion of patients with only one annual prescription was much higher in the BIFAP and Bavarian DB (34.7% and 29.2%) compared to the UK databases CPRD (14.1%) and THIN (14.5%) (Fig. 2a).

**Analysis of indication**. As shown in Table 2 for the 2008 data, significant differences were observed among the databases regarding the documented indications for the prescribing of a LABA-containing medication (p<0.0001). In all databases most patients were coded as “asthma” patients [without a COPD diagnosis] (between 36.2% and 52.2%). The proportion of COPD [without an asthma diagnosis] patients varied between 10.2% and 22.9%. Between 3.2% (Mondriaan–AHC) and 34.1% (Bavarian DB) of all patients receiving LABA-containing prescriptions were coded as having both “asthma and COPD”. The number of patients with “other and unknown” diagnoses was lowest in the UK databases (3.5% (THIN), 3.9% (CPRD)) and highest in the BIFAP database (27.6%).

### II. Stratification of results according to the three main indications

**Annual period prevalence rates**. With respect to the three patient strata (“asthma”, “COPD”, and “asthma and COPD”), the highest age- and sex-standardised PPR of patients with LABA-containing prescriptions was observed in the “asthma and COPD” stratum in all databases and all years (Fig. 1 b-d, details for 2008 are in Table 2). An increase in age- and sex-standardised PPR was observed in all three strata and all databases from 2002 onwards; however, the extent of the increase was different. In the “asthma” stratum, the PPR of the last year of the study period was between 1.7–2.0 times higher than in the first year of the study period; for the “COPD” stratum the PPR increased by 1.5–2.4 times in BIFAP and the two Dutch databases and by 6.2 and 7.6 times in the THIN and CPRD database.

**Number of LABA-containing prescriptions in 2008**. In all databases and in all three strata, most patients received 2–11 prescriptions per year. In all databases, the highest proportion of patients who received only 1 annual LABA-containing prescriptions was found in the “asthma” stratum whereas lowest proportion was found in the stratum “asthma and COPD” (Fig. 2 b-d).

## Discussion

By analysing seven databases from the electronic health records of five European countries with a standardised protocol, we found an increase in the prescribing of LABA-containing medications during the course of the study period. This increase was found in all patients with LABA-containing prescriptions as well as in patients diagnosed with asthma and, most notably, with COPD. During the study period, there were changes in asthma and COPD treatment guidelines, changes in the approved indications for using combined, fixed-dose ICS plus LABA products in COPD, and a regulatory warning issued regarding LABA use without ICS in asthma patients. These issues may have contributed to the changes over time found in our study and by others [27].

Despite the similar temporal trend in all databases, relevant inter-country differences in the prescribing prevalence rates for LABA-containing medications were discovered in our study. The differences among the databases were not reduced after standardising our results to the European reference population [23]. Thus, sex- and age-independent differences in LABA prescribing behaviour were confirmed among the five countries.

The TEDDY study, a pediatric asthma study, is one of the few studies that has examined LABA prescribing patterns by performing inter-country (Netherlands, Italy, and the UK) comparisons [12]. These investigators found results similar to ours, but there were methodological differences worth mentioning and limiting comparability. For example, in our study children were represented only by two age groups and we found in all databases higher PPRs of patients with LABA-containing prescriptions in children aged 10–19 years compared to 0–9 years. In the TEDDY study, an age-related increase was reported for LABA-ICS (fixed combination) for different age groups whereas data for LABA (regardless of ICS use) were not reported in detail [12]. Other “indirect” comparisons using national data [11] are of limited value due to different study periods and different data sources.

In our study, large differences were found between the two Dutch databases. The Mondriaan-AHC population is somewhat younger than the Mondriaan-NPCRD population. However, even after standardisation for age and sex, we still observed a higher rate of prescribing LABA-containing medications in Mondriaan-AHC as compared to Mondriaan-NPCRD. The main difference between the two databases is that Mondriaan-AHC also includes pharmacy medication dispensings from other specialties than GP in addition to GP prescriptions, whereas Mondriaan-NPCRD only covers GP prescriptions. Results for the two UK databases (CPRD and THIN) are more comparable due to the large overlap of the included practices (n = 327, resembling > 50%), and potentially due to the introduction of the Quality and Outcomes Framework, with its specifications regarding data collection and monitoring for asthma and COPD, during the study period [22].

Our inter-country analyses revealed a difference in the proportions of the main indications (asthma and/or COPD) for LABA-containing prescriptions. We found that patients diagnosed with asthma (“asthma” and “asthma and COPD”) accounted for 49.8% to 86.0% of patients prescribed a LABA-containing medication. A study out of the UK found that 76% of patients receiving a short- or long-acting bronchodilator had at least a diagnosis of asthma [28]. Nevertheless, the comparability of this prior study to our study is limited due to (1) the combined analysis of SABA (salbutamol, terbutalin and fenoterol) and the LABA compound salmeterol and (2) changes in prescribing habits over time since the patients in the UK study were included before 1994 and several changes to treatment guidelines have been made in the interval between the two study periods. Nevertheless, the predominance of diagnosed asthma, with and without concomitant COPD, in patients treated with LABA in the UK is indirectly confirmed in our study by a higher prevalence rate of asthma compared to the other countries. Indeed, in the UK and the Netherlands, the prevalence rates for “clinical asthma” were recently reported to be 18.2% and 15.3%, respectively, whereas lower values were reported for Denmark (10.2%), Germany (7.6%), and Spain (7.1%) [29].

An age-specific analysis of overall LABA prescribing is influenced by the age-specific prevalence rates of asthma and COPD. Consistent with this, we found two prevalence peaks (in children and in elderly patients) that were previously reported for bronchodilators (including SABA compounds) [28]. In our study, variations among the PPRs for LABA-containing medications in the different databases were less pronounced in younger patients compared to elderly patients. This may be attributable to pronounced differences in consultation patterns, as well as in prevalence rates of respiratory diseases in elderly patients between the countries included in our analysis. In addition to inter-country differences, intra-country differences as observed in other studies [28] may also have some influence on our results.

In two databases (BIFAP, Mondriaan-AHC), approximately one quarter of all patients receiving LABA-containing medication had neither a recorded diagnosis of asthma nor of COPD during the study period. One can hypothesize that these patients receiving LABA-containing drugs had a diagnosis of asthma or COPD recorded before the study period or that they were diagnosed with an acute bronchitis, which is not a labelled indication for LABA (i.e., off-label use). In future studies, these potential explanations should be examined in more detail.

According to the global initiatives for asthma and COPD and national guidelines of the countries included in this study, LABA-containing medication is recommended as controller medication [2, 3]. Nevertheless, the number of LABA-containing prescriptions per patient differed in our study to some extent between the countries and e.g. up to 30% of all asthma patients with a LABA-containing prescription received only one prescription. As shown in several studies, poor medication persistence with controller/maintenance drugs is an important issue in the long-term treatment of asthma and COPD [30, 31]. On the other hand, a small number of prescriptions might also be the result of other factors such as the more frequent re-assessment of patients’ symptoms in terms of high-quality guideline adherence. With respect to the initiation of LABA treatment, good guideline adherence has been confirmed in the UK [32]. Furthermore, variations in package size of LABA-containing medications between the countries might also contribute to the differences shown in our study.

### General limitations

In addition to the limitations stated above there are points which may have impacted this study. First, we estimated PPRs of patients with LABA-containing prescriptions irrespective of any other comedications (e.g. ICS) and clinical conditions (e.g. lung function parameters). Therefore, no statements regarding guideline adherence with respect to disease severity or combined drug therapy (e.g. concomitant use of LABA and ICS in asthma patients) can be made. In addition, by using drug prescription data (and not primarily indication data) for calculating PPRs, we were not able to calculate exact disease prevalence rates for asthma or COPD due to the non-inclusion of untreated persons or persons receiving other respiratory medications. Nevertheless, it has been shown that respiratory drug utilisation is a sufficient proxy for estimating disease prevalence rates [33]. Hence, estimation of disease prevalence rates by using LABA-containing prescription data might focus on patients diagnosed with more severe asthma and / or COPD. Second, we included data from the ambulatory sector only and thus other settings, such as hospitals, are not covered by our study. Third, since in most of the databases used in our study no direct linkage between drug prescription and indication is documented, all indications documented within the entire study period were used (static assessment of indication). Thus, a patient diagnosed with asthma first who later developed COPD was grouped into the “asthma and COPD” stratum. Since a significant number of (elderly) patients are suffering from both diseases, asthma and COPD (“Asthma-chronic obstructive pulmonary disease overlap syndrome (ACOS)”) [34, 35] a combined stratum “asthma and COPD” is justified. Furthermore, asthma and COPD are chronic diseases in most adults making a static assessment meaningful from a medical point of view. Nevertheless, over- as well as underestimation of indications influencing our study results cannot be ruled out. Fourth, there are uncertainties in the diagnosing of asthma and/or COPD under real life conditions that can result in misclassifications [36, 37]. Due to feasibility reasons and the primarily methodological character of our study, we abstained from validating documented indications but in all databases, regular routine data quality checks are performed. Fifth, non-COPD (acute) bronchitis, has a high prevalence rate in the young and in the elderly [28] and might be miscoded and registered as asthma in younger patients and as COPD in the elderly possibly influencing the results of our study. Sixth, since databases from different countries were included in our study, coding systems of diseases and drugs differs significantly. Despite a comprehensive and detailed mapping process, diagnoses and diseases might differ to some extent beyond the coding dictionaries we used to map them. Seventh, type of data (prescription data) and exclusion of prescriptions made by specialists were comparable for most databases. Nevertheless, for a few databases only dispensing data and data including prescriptions by specialists could be used influencing our study results. To sum up, the databases included in our study differ in several aspects. From a methodological point of view, this issue has been the main topic of interest in terms of how to overcome these differences. Nevertheless, several aspects mentioned above could not be fully addressed in this study and will limit the comparability of our results to some extent. In our study, comparability of results was improved by age- and sex standardisation of PPRs according to the European reference population and by using a standardised protocol.

## Conclusion

For the first time, we directly compared prescription data regarding LABA-containing drugs during the time period of 2002–2009 using seven databases from five Western European countries. Following a standardised protocol, we found an increasing trend for the period prevalence rate of patients with LABA-containing prescriptions in all five countries, and this was most pronounced in patients diagnosed with COPD even after age- and sex-standardisation. Despite some similarities in the PPRs of patients with LABA-containing prescriptions across countries, we uncovered several inter-country differences (e.g. number of prescriptions per patient in 12 months, prescription pattern in elderly patients). From a methodological perspective, we did confirm that our approach was a feasible tool for inter-country analyses of drug prescription data reflecting real-life conditions. Due to the standardized protocol, additional countries could be included in future studies.

## Supporting Information

S1 Table Exposure of interest. (DOCX)

S2 Table ICD-10 codes for indication. (DOCX)

S3 Table ICPC codes for indication. (DOCX)

S4 Table BIFAP specific ICPC coding. (DOCX)

S5 Table Read codes for indication. (DOCX)
