# Supplementary material for: Time Trends of Period Prevalence Rates of Patients with Inhaled Long-Acting Beta-2-Agonists-Containing Prescriptions: A European Comparative Database Study
Source: PLoS One. 2015 Feb 23;10(2):e0117628. doi: 10.1371/journal.pone.0117628 (PMC4338187; doi:10.1371/journal.pone.0117628)
Supplement: S1 Table — (DOCX) [file pone.0117628.s001.docx]

S1 Table: Exposure of interest

| **Drug class** | **ATC code** | **Compound name** |
| --- | --- | --- |
| Inhaled LABA | R03AC12 | Salmeterol |
|  | R03AC13 | Formoterol |
| Inhaled LABA combinations | R03AK06 | [Salmeterol and other drugs for obstructive airway diseases](http://www.whocc.no/atc_ddd_index/?code=R03AK06&showdescription=yes) |
|  | R03AK07 | [Formoterol and other drugs for obstructive airway diseases](http://www.whocc.no/atc_ddd_index/?code=R03AK07&showdescription=yes) |
|  | R03AK27 | Formoterol and Beclometasone |
|  | R03AK28 | Formoterol and Budesonide |
