# Supplementary material for: Time Trends of Period Prevalence Rates of Patients with Inhaled Long-Acting Beta-2-Agonists-Containing Prescriptions: A European Comparative Database Study
Source: PLoS One. 2015 Feb 23;10(2):e0117628. doi: 10.1371/journal.pone.0117628 (PMC4338187; doi:10.1371/journal.pone.0117628)
Supplement: S2 Table — (DOCX) [file pone.0117628.s002.docx]

S2 Table: ICD-10 codes for indication

| **Indication** | **ICD-10 Code** | **ICD-10 Term** |
| --- | --- | --- |
| Asthma | J45 | Asthma |
|  | J46 | Status asthmaticus |
| COPD | J44 | Other chronic obstructive pulmonary disease |
