# Supplementary material for: Time Trends of Period Prevalence Rates of Patients with Inhaled Long-Acting Beta-2-Agonists-Containing Prescriptions: A European Comparative Database Study
Source: PLoS One. 2015 Feb 23;10(2):e0117628. doi: 10.1371/journal.pone.0117628 (PMC4338187; doi:10.1371/journal.pone.0117628)
Supplement: S4 Table — (DOCX) [file pone.0117628.s004.docx]

S4 Table: BIFAP specific ICPC coding

| **Stratum** | **Codes / Term** |
| --- | --- |
| Asthma | R96 (Asthma) |
| COPD | BIFAP Thesaurus ICPC code “R79 Chronic Bronchitis”) OR BIFAP Thesaurus ICPC code “R91 Chronic Bronchitis/ Bronchiectasis” AND String text algorithms search (Spanish) identifying only Chronic Bronchitis (and NOT including Bronchiectasis)) OR BIFAP Thesaurus ICPC code “R95 Emphysema/ COPD”) |
