# Supplementary material for: Time Trends of Period Prevalence Rates of Patients with Inhaled Long-Acting Beta-2-Agonists-Containing Prescriptions: A European Comparative Database Study
Source: PLoS One. 2015 Feb 23;10(2):e0117628. doi: 10.1371/journal.pone.0117628 (PMC4338187; doi:10.1371/journal.pone.0117628)
Supplement: S5 Table — (DOCX) [file pone.0117628.s005.docx]

S5 Table: Read codes for indication

| **Stratum** | **Read Code** | **Read Term** |
| --- | --- | --- |
| Asthma | U60F600 | [X]ANTIASTHMATS CAUS ADVERSE EFFECTS IN THERAPEUT USE, NEC |
|  | 66YC.00 | ABSENT FROM WORK OR SCHOOL DUE TO ASTHMA |
|  | H333.00 | ACUTE EXACERBATION OF ASTHMA |
|  | TJF7z00 | ADVERSE REACTION TO ANTIASTHMATIC NOS |
|  | TJF7.00 | ADVERSE REACTION TO ANTIASTHMATICS |
|  | TJF7300 | ADVERSE REACTION TO THEOPHYLLINE (ASTHMA) |
|  | H330.11 | ALLERGIC ASTHMA |
|  | H33zz12 | ALLERGIC ASTHMA NEC |
|  | 1780.00 | ASPIRIN INDUCED ASTHMA |
|  | H33..00 | ASTHMA |
|  | G581.11 | ASTHMA - CARDIAC |
|  | 663j.00 | ASTHMA - CURRENTLY ACTIVE |
|  | 663h.00 | ASTHMA - CURRENTLY DORMANT |
|  | 663m.00 | ASTHMA ACCIDENT AND EMERGENCY ATTENDANCE SINCE LAST VISIT |
|  | 66YJ.00 | ASTHMA ANNUAL REVIEW |
|  | H33z100 | ASTHMA ATTACK |
|  | H33z111 | ASTHMA ATTACK NOS |
|  | 663t.00 | ASTHMA CAUSES DAYTIME SYMPTOMS 1 TO 2 TIMES PER MONTH |
|  | 663u.00 | ASTHMA CAUSES DAYTIME SYMPTOMS 1 TO 2 TIMES PER WEEK |
|  | 663v.00 | ASTHMA CAUSES DAYTIME SYMPTOMS MOST DAYS |
|  | 663r.00 | ASTHMA CAUSES NIGHT SYMPTOMS 1 TO 2 TIMES PER MONTH |
|  | 663N000 | ASTHMA CAUSING NIGHT WAKING |
|  | 9OJ..11 | ASTHMA CLINIC ADMINISTRATION |
|  | 8CR0.00 | ASTHMA CLINICAL MANAGEMENT PLAN |
|  | 1O2..00 | ASTHMA CONFIRMED |
|  | 8793.00 | ASTHMA CONTROL STEP 0 |
|  | 8794.00 | ASTHMA CONTROL STEP 1 |
|  | 8795.00 | ASTHMA CONTROL STEP 2 |
|  | 8796.00 | ASTHMA CONTROL STEP 3 |
|  | 8797.00 | ASTHMA CONTROL STEP 4 |
|  | 8798.00 | ASTHMA CONTROL STEP 5 |
|  | 663q.00 | ASTHMA DAYTIME SYMPTOMS |
|  | 663N.00 | ASTHMA DISTURBING SLEEP |
|  | 663N200 | ASTHMA DISTURBS SLEEP FREQUENTLY |
|  | 663N100 | ASTHMA DISTURBS SLEEP WEEKLY |
|  | 66YK.00 | ASTHMA FOLLOW-UP |
|  | 8CE2.00 | ASTHMA LEAFLET GIVEN |
|  | 663P.00 | ASTHMA LIMITING ACTIVITIES |
|  | 663x.00 | ASTHMA LIMITS WALKING ON THE FLAT |
|  | 663w.00 | ASTHMA LIMITS WALKING UP HILLS OR STAIRS |
|  | 663U.00 | ASTHMA MANAGEMENT PLAN GIVEN |
|  | 8B3j.00 | ASTHMA MEDICATION REVIEW |
|  | 9OJ4.00 | ASTHMA MONITOR 1ST LETTER |
|  | 9OJ5.00 | ASTHMA MONITOR 2ND LETTER |
|  | 9OJ6.00 | ASTHMA MONITOR 3RD LETTER |
|  | 9OJ3.00 | ASTHMA MONITOR OFFER DEFAULT |
|  | 9OJ8.00 | ASTHMA MONITOR PHONE INVITE |
|  | 9OJ7.00 | ASTHMA MONITOR VERBAL INVITE |
|  | 9OJA.11 | ASTHMA MONITORED |
|  | 663..11 | ASTHMA MONITORING |
|  | 9OJ..00 | ASTHMA MONITORING ADMIN. |
|  | 9OJZ.00 | ASTHMA MONITORING ADMIN.NOS |
|  | 66YR.00 | ASTHMA MONITORING BY DOCTOR |
|  | 66YQ.00 | ASTHMA MONITORING BY NURSE |
|  | 9OJA.00 | ASTHMA MONITORING CHECK DONE |
|  | 9OJ9.00 | ASTHMA MONITORING DELETED |
|  | 66YE.00 | ASTHMA MONITORING DUE |
|  | 663s.00 | ASTHMA NEVER CAUSES DAYTIME SYMPTOMS |
|  | 663O000 | ASTHMA NEVER DISTURBS SLEEP |
|  | 663f.00 | ASTHMA NEVER RESTRICTS EXERCISE |
|  | 66YP.00 | ASTHMA NIGHT-TIME SYMPTOMS |
|  | H33zz00 | ASTHMA NOS |
|  | 663O.00 | ASTHMA NOT DISTURBING SLEEP |
|  | 663Q.00 | ASTHMA NOT LIMITING ACTIVITIES |
|  | 9NI8.00 | ASTHMA OUTREACH CLINIC |
|  | 663W.00 | ASTHMA PROPHYLACTIC MEDICATION USED |
|  | 2126200 | ASTHMA RESOLVED |
|  | 212G.00 | ASTHMA RESOLVED |
|  | 663e.00 | ASTHMA RESTRICTS EXERCISE |
|  | 68C3.00 | ASTHMA SCREENING |
|  | 663e100 | ASTHMA SEVERELY RESTRICTS EXERCISE |
|  | 663V.00 | ASTHMA SEVERITY |
|  | 663e000 | ASTHMA SOMETIMES RESTRICTS EXERCISE |
|  | 663n.00 | ASTHMA TREATMENT COMPLIANCE SATISFACTORY |
|  | 663p.00 | ASTHMA TREATMENT COMPLIANCE UNSATISFACTORY |
|  | 178..00 | ASTHMA TRIGGER |
|  | H33z.00 | ASTHMA UNSPECIFIED |
|  | 9OJ1.00 | ATTENDS ASTHMA MONITORING |
|  | H334.00 | BRITTLE ASTHMA |
|  | H33..11 | BRONCHIAL ASTHMA |
|  | 66Y5.00 | CHANGE IN ASTHMA MANAGEMENT PLAN |
|  | H330.12 | CHILDHOOD ASTHMA |
|  | H312000 | CHRONIC ASTHMATIC BRONCHITIS |
|  | H47y000 | DETERGENT ASTHMA |
|  | 9N4Q.00 | DNA - DID NOT ATTEND ASTHMA CLINIC |
|  | 66YZ.00 | DOES NOT HAVE ASTHMA MANAGEMENT PLAN |
|  | 8H2P.00 | EMERGENCY ADMISSION, ASTHMA |
|  | 663d.00 | EMERGENCY ASTHMA ADMISSION SINCE LAST APPOINTMENT |
|  | 9hA2.00 | EXCEPTED FROM ASTHMA QUALITY INDICATORS: INFORMED DISSENT |
|  | 9hA1.00 | EXCEPTED FROM ASTHMA QUALITY INDICATORS: PATIENT UNSUITABLE |
|  | 9hA..00 | EXCEPTION REPORTING: ASTHMA QUALITY INDICATORS |
|  | 173A.00 | EXERCISE INDUCED ASTHMA |
|  | H33zz11 | EXERCISE INDUCED ASTHMA |
|  | H330.00 | EXTRINSIC (ATOPIC) ASTHMA |
|  | H330z00 | EXTRINSIC ASTHMA NOS |
|  | H330111 | EXTRINSIC ASTHMA WITH ASTHMA ATTACK |
|  | H330100 | EXTRINSIC ASTHMA WITH STATUS ASTHMATICUS |
|  | H330000 | EXTRINSIC ASTHMA WITHOUT STATUS ASTHMATICUS |
|  | 8791.00 | FURTHER ASTHMA - DRUG PREVENT. |
|  | 14B4.00 | H/O: ASTHMA |
|  | H330.13 | HAY FEVER WITH ASTHMA |
|  | H330011 | HAY FEVER WITH ASTHMA |
|  | 679J.00 | HEALTH EDUCATION - ASTHMA |
|  | H331.00 | INTRINSIC ASTHMA |
|  | H331z00 | INTRINSIC ASTHMA NOS |
|  | H331111 | INTRINSIC ASTHMA WITH ASTHMA ATTACK |
|  | H331100 | INTRINSIC ASTHMA WITH STATUS ASTHMATICUS |
|  | H331000 | INTRINSIC ASTHMA WITHOUT STATUS ASTHMATICUS |
|  | H331.11 | LATE ONSET ASTHMA |
|  | H33z200 | LATE-ONSET ASTHMA |
|  | 663V100 | MILD ASTHMA |
|  | H332.00 | MIXED ASTHMA |
|  | 663V200 | MODERATE ASTHMA |
|  | 663y.00 | NUMBER OF ASTHMA EXACERBATIONS IN PAST YEAR |
|  | 663V000 | OCCASIONAL ASTHMA |
|  | 173c.00 | OCCUPATIONAL ASTHMA |
|  | 9Q21.00 | PATIENT IN ASTHMA STUDY |
|  | H330.14 | POLLEN ASTHMA |
|  | 8HTT.00 | REFERRAL TO ASTHMA CLINIC |
|  | 9OJ2.00 | REFUSES ASTHMA MONITORING |
|  | 9N1d.00 | SEEN IN ASTHMA CLINIC |
|  | H35y600 | SEQUOIOSIS (RED-CEDAR ASTHMA) |
|  | 663V300 | SEVERE ASTHMA |
|  | H33z011 | SEVERE ASTHMA ATTACK |
|  | H33z000 | STATUS ASTHMATICUS NOS |
|  | 66YA.00 | STEP DOWN CHANGE IN ASTHMA MANAGEMENT PLAN |
|  | 66Y9.00 | STEP UP CHANGE IN ASTHMA MANAGEMENT PLAN |
|  | H35y700 | WOOD ASTHMA |
|  | 173d.00 | WORK AGGRAVATED ASTHMA |
| COPD | 66YB.00 | CHRONIC OBSTRUCTIVE PULMONARY DISEASE MONITORING |
|  | 66YD.00 | CHRONIC OBSTRUCTIVE PULMONARY DISEASE MONITORING DUE |
|  | 66Yd.00 | COPD ACCIDENT AND EMERGENCY ATTENDANCE SINCE LAST VISIT |
|  | 66Ye.00 | EMERGENCY COPD ADMISSION SINCE LAST APPOINTMENT |
|  | 66Yf.00 | NUMBER OF COPD EXACERBATIONS IN PAST YEAR |
|  | 66Yg.00 | CHRONIC OBSTRUCTIVE PULMONARY DISEASE DISTURBS SLEEP |
|  | 66Yh.00 | CHRONIC OBSTRUCTIVE PULMONARY DISEASE DOES NOT DISTURB SLEEP |
|  | 66YI.00 | COPD SELF-MANAGEMENT PLAN GIVEN |
|  | 66Yi.00 | MULTIPLE COPD EMERGENCY HOSPITAL ADMISSIONS |
|  | 66YL.00 | CHRONIC OBSTRUCTIVE PULMONARY DISEASE FOLLOW-UP |
|  | 66YL.11 | COPD FOLLOW-UP |
|  | 66YM.00 | CHRONIC OBSTRUCTIVE PULMONARY DISEASE ANNUAL REVIEW |
|  | 66YS.00 | CHRONIC OBSTRUCTIVE PULMONARY DISEASE MONITORING BY NURSE |
|  | 66YT.00 | CHRONIC OBSTRUCTIVE PULMONARY DISEASE MONITORING BY DOCTOR |
|  | 8CR1.00 | CHRONIC OBSTRUCTIVE PULMONARY DISEASE CLINI MANAGEMENT PLAN |
|  | 8H2R.00 | ADMIT COPD EMERGENCY |
|  | 9h5..00 | EXCEPTION REPORTING: COPD QUALITY INDICATORS |
|  | 9h51.00 | EXCEPTED FROM COPD QUALITY INDICATORS: PATIENT UNSUITABLE |
|  | 9h52.00 | EXCEPTED FROM COPD QUALITY INDICATORS: INFORMED DISSENT |
|  | 9kf0.00 | COPD PATIENT UNSUITABLE FOR PULMONARY REHAB - ENH SERV ADMIN |
|  | 9Oi..00 | CHRONIC OBSTRUCTIVE PULMONARY DISEASE MONITORING ADMIN |
|  | 9Oi0.00 | CHRONIC OBSTRUCTIVE PULMONARY DISEASE MONITORING 1ST LETTER |
|  | 9Oi1.00 | CHRONIC OBSTRUCTIVE PULMONARY DISEASE MONITORING 2ND LETTER |
|  | 9Oi2.00 | CHRONIC OBSTRUCTIVE PULMONARY DISEASE MONITORING 3RD LETTER |
|  | 9Oi3.00 | CHRONIC OBSTRUCTIVE PULMONARY DISEASE MONITORING VERB INVITE |
|  | 9Oi4.00 | CHRONIC OBSTRUCTIVE PULMONARY DISEASE MONITOR PHONE INVITE |
|  | H3...00 | CHRONIC OBSTRUCTIVE PULMONARY DISEASE |
|  | H3...11 | CHRONIC OBSTRUCTIVE AIRWAYS DISEASE |
|  | H31..00 | CHRONIC BRONCHITIS |
|  | H310.00 | SIMPLE CHRONIC BRONCHITIS |
|  | H310000 | CHRONIC CATARRHAL BRONCHITIS |
|  | H310z00 | SIMPLE CHRONIC BRONCHITIS NOS |
|  | H311.00 | MUCOPURULENT CHRONIC BRONCHITIS |
|  | H311000 | PURULENT CHRONIC BRONCHITIS |
|  | H311100 | FETID CHRONIC BRONCHITIS |
|  | H311z00 | MUCOPURULENT CHRONIC BRONCHITIS NOS |
|  | H312.00 | OBSTRUCTIVE CHRONIC BRONCHITIS |
|  | H312000 | CHRONIC ASTHMATIC BRONCHITIS |
|  | H312011 | CHRONIC WHEEZY BRONCHITIS |
|  | H312100 | EMPHYSEMATOUS BRONCHITIS |
|  | H312200 | ACUTE EXACERBATION OF CHRONIC OBSTRUCTIVE AIRWAYS DISEASE |
|  | H312300 | BRONCHIOLITIS OBLITERANS |
|  | H312z00 | OBSTRUCTIVE CHRONIC BRONCHITIS NOS |
|  | H313.00 | MIXED SIMPLE AND MUCOPURULENT CHRONIC BRONCHITIS |
|  | H31y.00 | OTHER CHRONIC BRONCHITIS |
|  | H31y100 | CHRONIC TRACHEOBRONCHITIS |
|  | H31yz00 | OTHER CHRONIC BRONCHITIS NOS |
|  | H31z.00 | CHRONIC BRONCHITIS NOS |
|  | H32..00 | EMPHYSEMA |
|  | H320.00 | CHRONIC BULLOUS EMPHYSEMA |
|  | H320000 | SEGMENTAL BULLOUS EMPHYSEMA |
|  | H320100 | ZONAL BULLOUS EMPHYSEMA |
|  | H320200 | GIANT BULLOUS EMPHYSEMA |
|  | H320300 | BULLOUS EMPHYSEMA WITH COLLAPSE |
|  | H320z00 | CHRONIC BULLOUS EMPHYSEMA NOS |
|  | H321.00 | PANLOBULAR EMPHYSEMA |
|  | H322.00 | CENTRILOBULAR EMPHYSEMA |
|  | H32y.00 | OTHER EMPHYSEMA |
|  | H32y000 | ACUTE VESICULAR EMPHYSEMA |
|  | H32y100 | ATROPHIC (SENILE) EMPHYSEMA |
|  | H32y111 | ACUTE INTERSTITIAL EMPHYSEMA |
|  | H32y200 | MACLEOD'S UNILATERAL EMPHYSEMA |
|  | H32yz00 | OTHER EMPHYSEMA NOS |
|  | H32yz11 | SAWYER - JONES SYNDROME |
|  | H32z.00 | EMPHYSEMA NOS |
|  | H36..00 | MILD CHRONIC OBSTRUCTIVE PULMONARY DISEASE |
|  | H37..00 | MODERATE CHRONIC OBSTRUCTIVE PULMONARY DISEASE |
|  | H38..00 | SEVERE CHRONIC OBSTRUCTIVE PULMONARY DISEASE |
|  | H39..00 | VERY SEVERE CHRONIC OBSTRUCTIVE PULMONARY DISEASE |
|  | H3y..00 | OTHER SPECIFIED CHRONIC OBSTRUCTIVE AIRWAYS DISEASE |
|  | H3y..11 | OTHER SPECIFIED CHRONIC OBSTRUCTIVE PULMONARY DISEASE |
|  | H3y0.00 | CHRONIC OBSTRUCT PULMONARY DIS WITH ACUTE LOWER RESP INFECTN |
|  | H3y1.00 | CHRON OBSTRUCT PULMONARY DIS WTH ACUTE EXACERBATION, UNSPEC |
|  | H3z..00 | CHRONIC OBSTRUCTIVE AIRWAYS DISEASE NOS |
|  | H3z..11 | CHRONIC OBSTRUCTIVE PULMONARY DISEASE NOS |
|  | H464000 | CHRONIC EMPHYSEMA DUE TO CHEMICAL FUMES |
|  | H581.00 | INTERSTITIAL EMPHYSEMA |
|  | H582.00 | COMPENSATORY EMPHYSEMA |
|  | Hyu3.00 | [X]CHRONIC LOWER RESPIRATORY DISEASES |
|  | Hyu3000 | [X]OTHER EMPHYSEMA |
|  | Hyu3100 | [X]OTHER SPECIFIED CHRONIC OBSTRUCTIVE PULMONARY DISEASE |
|  | SK07.00 | SUBCUTANEOUS EMPHYSEMA |
|  | SP2y000 | SURGICAL EMPHYSEMA |
